# Comparing Postoperative Outcomes Between Laparoscopic-Assisted Percutaneous Internal Ring Suturing and Laparoscopic Intracorporeal Internal Ring Suturing in the Management of Paediatric Inguinal Herniae: A Retrospective Review in Jordan

**DOI:** 10.7759/cureus.95131

**Published:** 2025-10-22

**Authors:** Zakaria W Shkoukani, Tara Ghazi, Jude Foudeh, Dalia Kaadan, Karam Darwish, Yazan D Abualnadi, Yumen Sejari, Dana Al-Qudah, Bashar Al-Shboul, Raed Al-Taher

**Affiliations:** 1 Department of Urology, Mersey and West Lancashire Teaching Hospitals NHS Trust, Prescot, GBR; 2 Department of General Surgery, Jordan University Hospital, Amman, JOR; 3 School of Information Technology, University of Jordan, Amman, JOR

**Keywords:** intracorporeal internal ring suturing, laparoscopic assisted hernia repair, paediatric inguinal hernia repair, percutaneous internal ring suturing, postoperative outcomes

## Abstract

Background: Congenital inguinal herniae are among the most common conditions requiring surgery in children. While laparoscopic repair offers diagnostic and cosmetic advantages over open repair, debate persists regarding the optimal method of internal ring closure. This study compares outcomes of laparoscopic intracorporeal internal ring suturing (LICS) and laparoscopic-assisted percutaneous internal ring suturing (LAPIRS) in a paediatric population.

Methods: We conducted a retrospective cohort study of children who underwent laparoscopic inguinal hernia repair at Jordan University Hospital between January 2019 and December 2020. Demographic data, clinical presentation, operative details, complications, costs, and follow-up outcomes were analysed. Patients undergoing concurrent orchidopexy were excluded.

Results: Of the 221 patients treated for inguinal herniae, 38 met the inclusion criteria (27 males, 11 females; mean age 3 years, range 45 days to 13 years). Seventeen patients (44.7%) underwent LAPIRS and 21 patients (55.3%) underwent LICS. No intraoperative complications occurred. Mean operative time was 45 minutes, with no significant difference between techniques or laterality (p = 0.849). Postoperative morbidity was minimal, with only one case of umbilical disfigurement following LAPIRS. No recurrences or wound infections were observed after a minimum of six months’ follow-up. The median cost was 512 Jordanian Dinars (JOD), equivalent to $720 US dollars, and was unaffected by technique (p = 0.395).

Conclusions: Both LICS and LAPIRS are safe, effective, and cosmetically favourable options for paediatric inguinal hernia repair. Outcomes were comparable, with negligible morbidity and no recurrences. Given potential differences in learning curve, cosmesis, and cost reported internationally, larger prospective studies with longer follow-up are warranted to refine best-practice recommendations.

## Introduction

Inguinal hernia, defined as the protrusion of intra-abdominal contents through the abdominal wall, can occur either indirectly through a patent processus vaginalis (PPV) or directly through the anterior abdominal wall. Complications associated with this condition include incarceration, strangulation, and bowel obstruction, among others. Inguinal herniae are prevalent across both paediatric and adult populations, and minimising the risk of these complications is crucial to the management of the condition in all age groups [1].

Surgical intervention remains the gold standard for the management of inguinal herniae, with repair being one of the most commonly performed surgical procedures worldwide [2]. Congenital inguinal herniae, occurring in approximately 1-5% of the paediatric population, are among the most frequent surgical conditions in children [3].

In paediatric cases, a PPV is typically the primary aetiological factor for congenital indirect inguinal herniae, whereas direct abdominal wall herniations are less common [1]. Traditionally, such herniae have been managed through open surgical techniques. However, recent advancements in surgical technology have led to the increased adoption of minimally invasive approaches, particularly laparoscopy. The choice of surgical technique often depends on the surgeon’s expertise and available resources [4].

The pioneering work of Misra et al., who first successfully performed laparoscopic repair of an indirect inguinal hernia in a child, marked a significant advancement in paediatric hernia surgery and laid the foundation for the broader application of laparoscopic techniques [5]. While open hernia repair remains a widely used and effective approach, laparoscopic methods are gaining favour due to their favourable postoperative outcomes, including reduced pain, shorter recovery times, and lower complication rates [2].

Various techniques exist for the suturing of the internal inguinal ring during laparoscopic hernia repair. Laparoscopic intracorporeal internal ring suturing (LICS) is the traditional method, but laparoscopic-assisted percutaneous internal ring suturing (LAPIRS), a less invasive alternative, has also been shown to be safe and effective [6]. Introduced by Polish surgeon Patkowski in 2004, LAPIRS requires only a single umbilical port and a needle puncture, offering a more minimally invasive approach to hernia repair [2].

In Jordan, there is a notable lack of research focusing on laparoscopic hernia repair in the paediatric population. To our knowledge, only one study from the Middle East, conducted by Ali et al. in Egypt, has compared LAPIRS with traditional laparoscopic herniotomy in children, concluding that LAPIRS is a safe, simple, and minimally invasive technique [7]. This study aims to address the existing research gap by comparing postoperative outcomes of two laparoscopic techniques, LAPIRS and laparoscopic intracorporeal suturing (LICS), at a tertiary care centre. Specifically, the study will evaluate outcomes related to postoperative recovery, cost-effectiveness, and time efficiency.

## Materials and methods

Study design

This study employed a retrospective cohort design to evaluate the efficacy and postoperative outcomes of two laparoscopic techniques: LAPIRS and LICS in the repair of inguinal herniae in paediatric patients. The study was conducted in the Division of Paediatric Surgery at Jordan University Hospital, a tertiary care centre, over a two-year period from January 2019 to December 2020.

Data collection

Data for this study were obtained from the electronic medical records system at Jordan University Hospital, which houses comprehensive patient records. The following variables were systematically collected: demographic information such as age, sex, weight, and relevant medical history; details of the clinical presentation, including the nature of the initial presentation (for example, asymptomatic, pain, or swelling) and the laterality (right or left side) of the hernia; and information on any radiological investigations, including ultrasound or other imaging modalities, along with their findings. Additionally, data were gathered regarding the surgical technique employed (LAPIRS or LICS), operative time, intraoperative findings, and any challenges encountered during surgery. Postoperative complications, such as infection, recurrence, or injury to adjacent structures, were also documented, along with the length of hospital stay and the time to mobilisation or discharge. In cases where data were incomplete, the parents or guardians of the patients were contacted by telephone to obtain any missing information.

Inclusion and exclusion criteria

The study included paediatric patients aged between birth and 14 years who underwent laparoscopic inguinal hernia repair at Jordan University Hospital between January 2019 and December 2020. Patients who had concurrent ipsilateral inguinal hernia repair and orchidopexy in the same operative setting were excluded to avoid confounding the results. Additionally, cases involving bilateral inguinal hernia repairs performed simultaneously during the same surgical session were also excluded.

Statistical analysis

Data were organised and entered into a Microsoft Excel spreadsheet (Microsoft Corporation, Redmond, Washington) for preliminary analysis. Statistical analyses were conducted using the IBM SPSS Statistics for Windows, Version 25 (Released 2017; IBM Corp., Armonk, New York). Patients in the two groups (LAPIRS and LICS) were matched by age to control for age-related variability in outcomes. Postoperative outcomes, including operative time, complication rates, and recovery metrics, were compared using multiple regression analysis to adjust for potential confounding variables. Operative time was analysed using a two-way analysis of variance (technique × laterality), while overall cost was compared between techniques using a linear regression model equivalent to a two-sample t-test. A p-value of less than 0.05 was considered statistically significant.

Ethical approval

Ethical approval for the study was obtained from the Institutional Review Board (IRB) of Jordan University Hospital (Ref: 1012021/3182). Given the retrospective nature of the study, verbal informed consent was sought from the parents or guardians of all patients included in the study. All patient data were anonymised and handled in compliance with the ethical standards governing medical research.

Technical details of the surgical procedures

For the LAPIRS technique, a 5-mm 30-degree laparoscope was inserted through an umbilical trocar, which was introduced using an open technique. Following abdominal insufflation, a 2-mm incision was made at the lateral edge of the inguinal crease, and a 2-0 or 3-0 polydioxanone (PDS) suture (Ethicon, UK) was used to create a purse-string suture through the skin under direct visualisation with the laparoscope. The PPV was then closed at the level of the internal inguinal ring, while preserving the vas deferens and gonadal vessels in male patients and incorporating the round ligament in female patients. The knot was buried under the skin, and the umbilical trocar was removed. The fascia was closed using the same suture material, and skin closure was achieved with sterile strips. The abdominal cavity was deflated at the conclusion of the procedure.

For the LICS technique, a 5-mm 30-degree laparoscope was again introduced through an umbilical trocar, and the abdominal cavity was insufflated. Two additional 3 mm trocars were placed in the right and left lower quadrants, approximately 1-2 cm below the level of the umbilicus. The PPV was sutured intracorporeally at the level of the internal inguinal ring using 2-0 or 3-0 Ethibond Excel suture material (Ethicon, UK). As with the LAPIRS technique, the vas deferens and gonadal vessels were preserved in males, and the round ligament was incorporated in females. After the procedure, the side trocars were removed, and the abdominal cavity was deflated. The fascia was closed with the same suture material, and the skin incision was approximated using sterile strips.

Both procedures were performed under general anaesthesia, with patients monitored closely for immediate postoperative complications. Recovery protocols followed standard practice for laparoscopic surgery, and patients were discharged once they had recovered sufficiently and met discharge criteria.

## Results

Patient demographics

During the study period (January 2019 to December 2020), a total of 221 paediatric patients underwent inguinal hernia repair at Jordan University Hospital. Of these, 183 were excluded as they underwent open repair or combined orchidopexy, leaving 38 patients eligible for analysis.

The study cohort consisted of 27 males (71.1%) and 11 females (28.9%), yielding a male-to-female ratio of 2.5:1. The mean age at surgery was 3.0 ± 3.2 years (range, 45 days to 13 years). When stratified by age group, the majority of herniae were observed in children aged 6 years or older (28.9%, n = 11), followed by toddlers aged 1 to 3 years (26.3%, n = 10), preschool children aged 3 to 6 years (21.1%, n = 8), and infants younger than 1 year (23.7%, n = 9).

Associated conditions were uncommon. Three patients (7.9%) had a history of previously repaired undescended testes, and one (2.6%) had an abdominal wall defect. No patients had neuromuscular, metabolic, or connective tissue disorders (Table 1).

**Table 1:** Patient demographics and associated conditions. Data are presented as number (n), with percentages in parentheses (%).

Variable	Category	n (%)
Age groups	Infants (<1 year)	9 (23.7)
	Toddlers (1-3 years)	10 (26.3)
	Preschool (3-6 years)	8 (21.1)
	≥6 years	11 (28.9)
	Total	38 (100)
Gender	Male	27 (71.1)
	Female	11 (28.9)
	Total	38 (100)
Associated conditions	Undescended testes (previously repaired)	3 (7.9)
	Abdominal wall defect	1 (2.6)
	Other congenital/systemic conditions	0 (0)

Preoperative assessment

Most patients (87%, n = 33) presented in an elective setting, whereas 13% (n = 5) required emergency intervention. Among elective cases, LICS was employed more frequently (60.6%, n = 20) compared with LAPIRS (39.4%, n = 13). Conversely, in emergency cases, LAPIRS was the predominant approach (80%, n = 4 vs 20%, n = 1 for LICS), primarily due to its perceived advantages in the emergency setting, including shorter operating times, reduced instrumentation requirements, and surgeon preference.

Preoperative laterality assessment showed right-sided herniae in 21 patients (55.3%), left-sided herniae in 11 patients (28.9%), and bilateral herniae in 6 patients (15.8%). The vast majority of patients were diagnosed preoperatively with a simple inguinal hernia (92.1%, n = 35). Two patients (5.3%) had incarcerated herniae, all successfully reduced prior to repair, and one patient (2.6%) had a hydrocoele. Stratification by age group and sex is presented in Table 2.

**Table 2:** Preoperative clinical characteristics. Data are presented as number (n), with percentages in parentheses (%).

Variable	Category	Age groups				Gender	
		Infants (<1 year)	Toddlers (1-3 years)	Preschool (3-6 years)	≥6 years	Male	Female
Presentation setting	Elective	7 (21.2)	8 (24.2)	7 (21.2)	11 (33.3)	22 (66.7)	11 (33.3)
	Emergency	2 (40.0)	2 (40.0)	1 (20.0)	0 (0.0)	5 (100.0)	0 (0.0)
Total		9 (23.7)	10 (26.3)	8 (21.1)	11 (28.9)	27 (71.1)	11 (28.9)
Preoperative side	Left inguinal hernia	2 (18.2)	3 (27.3)	2 (18.2)	4 (36.4)	8 (72.7)	3 (27.3)
	Right inguinal hernia	5 (23.8)	6 (28.6)	3 (14.3)	7 (33.3)	15 (71.4)	6 (28.6)
	Bilateral herniae	2 (33.3)	1 (16.7)	3 (50.0)	0 (0.0)	4 (66.7)	2 (33.3)
Total		9 (23.7)	10 (26.3)	8 (21.1)	11 (28.9)	27 (71.1)	11 (28.9)
Preoperative diagnosis	Hydrocele	0 (0.0)	1 (100.0)	0 (0.0)	0 (0.0)	1 (100.0)	0 (0.0)
	Simple inguinal hernia	8 (22.9)	9 (25.7)	7 (20.0)	11 (31.4)	24 (68.6)	11 (31.4)
	Incarcerated inguinal hernia	1 (50.0)	0 (0.0)	1 (50.0)	0 (0.0)	2 (100.0)	0 (0.0)
Total		9 (23.7)	10 (26.3)	8 (21.1)	11 (28.9)	27 (71.1)	11 (28.9)

Intraoperative findings and surgical technique

Laparoscopic exploration revealed a notable incidence of synchronous contralateral PPV: 36.4% (n = 4) of patients with preoperative left herniae were found to have a contralateral PPV, and 28.6% (n = 6) of patients with right herniae had a contralateral PPV. Overall, 54 PPVs were repaired (27 unilateral and 27 bilateral).

**Figure 1:**
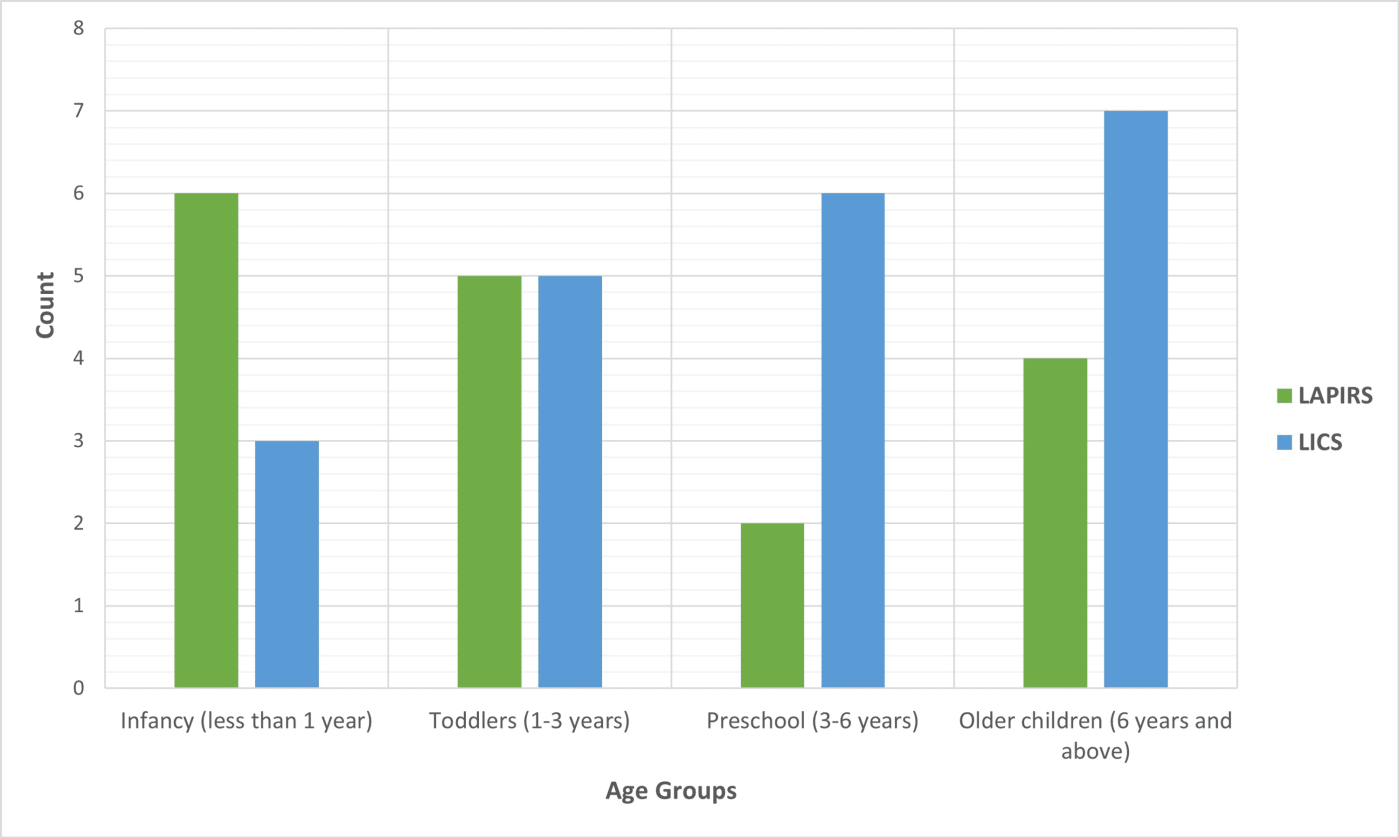
Distribution of surgical technique across age groups. Bar chart showing the distribution of laparoscopic repair technique by patient age group. Data are presented as number (n), representing case counts and the technique performed across different age groups. Green bars represent patients who underwent laparoscopic-assisted percutaneous internal ring suturing (LAPIRS). Blue bars represent patients who underwent laparoscopic intracorporeal internal ring suturing (LICS). Age groups are stratified as: Infants (<1 year), Toddlers (1–3 years), Preschool (3–6 years), and ≥6 years. The two techniques were used across all age categories, with relatively even distribution between groups.

Seventeen patients (23 PPVs, 44.7%) underwent LAPIRS, while 21 patients (31 PPVs, 55.3%) underwent LICS. The age distribution between the two groups was similar, as shown in Figure 1.

No intraoperative complications (e.g., vascular injury, visceral injury, or bleeding) were reported in either technique group.

Operative time

The mean operative time for the cohort was 45 minutes (range: 20-60 minutes). Mean time was comparable between techniques: 45.0 ± 8.2 minutes for unilateral LICS, 47.0 ± 9.1 minutes for bilateral LICS, 45.5 ± 7.9 minutes for unilateral LAPIRS, and 48.3 ± 10.2 minutes for bilateral LAPIRS. A two-way analysis of variance (technique × laterality) showed no significant effect of surgical technique on operative time (F(1,36) = 0.037, p = 0.849).

**Table 3:** Mean operating time by technique and laterality. Data are presented as number (n), with percentages in parentheses (%) for cases. Operating time is expressed as mean ± standard deviation (SD) in minutes. A p-value of <0.05 is considered statistically significant. LICS: laparoscopic intracorporeal internal ring suturing, LAPIRS: laparoscopic assisted percutaneous internal ring suturing.

Technique	Laterality	n (%)	Mean time ± SD (min)	p-value
LICS	Unilateral	11 (29.0)	45.0 ± 8.2	
LICS	Bilateral	10 (26.0)	47.0 ± 9.1	
LAPIRS	Unilateral	11 (29.0)	45.5 ± 7.9	
LAPIRS	Bilateral	6 (16.0)	48.3 ± 10.2	
Total		38 (100.0)	45.7 ± 8.8	0.849

Post-operative assessment and follow-up

All patients were followed up for a minimum of six months according to a standardised protocol. Follow-up was conducted exclusively through the paediatric surgery outpatient clinic, with no assessments performed via telephone. The schedule consisted of an initial review at two weeks postoperatively to evaluate the patient’s general condition and inspect the surgical wounds for signs of infection. Additional assessments were conducted at three and six months to monitor for postoperative complications and hernia recurrence.

Hernia recurrence was defined as any reappearance of an inguinal bulge confirmed by both physical examination and ultrasonography. No recurrences were identified in either the LAPIRS or LICS groups during the follow-up period. Wound infections were diagnosed based on clinical findings, including erythema, swelling, discharge, or wound dehiscence, supported by positive microbiological cultures from wound swabs in accordance with CDC criteria. One patient (2.6%) in the LAPIRS group developed mild umbilical disfigurement, which was managed conservatively (Clavien-Dindo classification Grade I). No cases of wound infection, port-site herniae, chronic groin pain, or testicular atrophy were observed.

Only two patients (5.3%) had missing information, prompting the research team to contact the parents or guardians for clarification.

The median procedural cost was 512 Jordanian Dinars (JOD) (range, 152-720 JOD), equivalent to $721 US dollars (range, $214-$1014). Comparison of overall cost between techniques showed no significant difference (linear regression coefficient test: t(36) = 0.86, p = 0.395; equivalently F(1, 36) = 0.74, p = 0.395).

## Discussion

The management of paediatric inguinal herniae has evolved substantially with the advent of minimally invasive surgery. Laparoscopy provides superior visualisation of the internal ring and contralateral internal inguinal ring (PPV), allows simultaneous identification and repair of occult contralateral defects, and generally yields improved cosmetic outcomes compared with traditional open repair [2,6]. Despite these advantages, there is ongoing debate regarding the optimal laparoscopic technique for internal ring closure, principally between LICS and LAPIRS, also referred to in some series as percutaneous extraperitoneal closure or LPEC.

Most published series have compared laparoscopic approaches with the conventional open technique rather than directly comparing intracorporeal and percutaneous laparoscopic methods [8]. Comparative data directly pitting LICS against LAPIRS remain relatively scarce, with Wang et al. providing one of the more recent head-to-head analyses [9]. In their series, Wang et al. reported a slightly higher (but statistically non-significant) rate of intraoperative bleeding in the intracorporeal group; importantly, bleeding was clinically minimal in both approaches and did not necessitate conversion to open surgery [9]. Our cohort mirrors these findings: intraoperative bleeding was negligible across both techniques, and no major intraoperative complications were encountered, supporting the safety of both LICS and LAPIRS in experienced hands.

Operative time is frequently cited when selecting a technique. Wang et al. reported significantly shorter operative times with the percutaneous method, a finding commonly attributed to avoidance of intracorporeal knot-tying, which is technically more demanding and time-consuming [9]. Learning-curve studies also favour the percutaneous approach: Barroso et al. demonstrated more rapid attainment of proficiency with percutaneous techniques compared with intracorporeal suturing, making LAPIRS an attractive option in training environments [10]. Timberlake and colleagues also highlighted the relative simplicity and reproducibility of the percutaneous technique in practical paediatric surgery [11]. In our series, however, we found no statistically significant difference in operative duration between the two techniques, and all repairs were completed within 60 minutes. This likely reflects a combination of factors, including institutional experience with both techniques, case selection, and the modest sample size, which limits power to detect small differences. Additionally, our institution provides similar training opportunities and supervision levels for both approaches, which may contribute to the comparable operative times observed.

Recurrence is the most consequential long-term outcome for hernia repair. Wang et al. observed more recurrences in the intracorporeal group than in the percutaneous group (4 vs 1), although this difference did not reach statistical significance [9]. Larger series and registry data report recurrence rates for percutaneous methods at the lower end of the spectrum (near 0-0.4% in some series) [11,12], whereas reported recurrence rates for intracorporeal suturing have varied more widely (0.4-4.1% in selected series) and may be influenced by technical factors such as knot tension and completeness of purse-string closure [13,14]. In our study, there were no recurrences in either group during a minimum follow-up of six months. While reassuring, this finding must be interpreted cautiously because longer follow-up and larger cohorts are necessary to definitively compare recurrence risk between techniques.

Postoperative morbidity in our cohort was minimal. Only a single patient developed minor umbilical disfigurement after LAPIRS; there were no wound infections, port-site herniae, hydrocoeles, or testicular atrophy. These observations align with previously reported low morbidity for both techniques [9,12]. Wang et al. reported a slightly higher (non-significant) surgical-site infection rate in the intracorporeal group [9], and Miyake et al.’s large Japanese series documented a small number of infections confined to the umbilical incision after percutaneous repair (0.78%) [12]. Taken together, the literature supports low overall complication rates for both approaches when performed with attention to atraumatic technique and preservation of the vas deferens and gonadal vessels.

Cosmetic outcome is an increasingly important consideration in paediatric surgery, particularly for elective cases. LAPIRS typically requires a single umbilical port plus a small percutaneous puncture, whereas LICS commonly requires three ports. Consequently, LAPIRS is often perceived to offer superior cosmesis [2,6,10,15]. This advantage, along with the shorter learning curve and potentially lower instrument needs, likely contributes to the growing adoption of percutaneous approaches in many centres.

Cost considerations may also influence technique selection. The percutaneous method can be less resource-intensive due to fewer trocars and simpler instrumentation, potentially translating to lower procedure costs in some settings [16,17]. In our cohort, the median cost did not differ significantly between groups; however, cost analyses are highly sensitive to local pricing structures, sterile instrument re-use policies, and whether disposables or advanced laparoscopic tools are employed. A formal cost-effectiveness analysis in a larger sample would be required to draw firm conclusions.

The financial cost of laparoscopic inguinal hernia repair is known to be context-dependent, varying significantly between institutions, regions, and healthcare systems. In our study, the average cost per procedure was estimated based on hospital billing data. While this provides a general estimate, we acknowledge that a detailed itemisation of individual cost components, such as trocars, instruments, anaesthesia duration, and disposables, was not available. Additionally, the analysis does not account for the specific payer perspective (for example, public versus private healthcare or insurance reimbursement), which may influence overall cost implications. Therefore, although no significant cost difference was observed between the LAPIRS and LICS groups in our setting, this conclusion should be interpreted with caution given these limitations. Future studies incorporating granular cost breakdowns and standardised economic evaluation frameworks are warranted to enable more definitive comparisons.

Strengths of our study include the use of a single tertiary-care institutional dataset with complete perioperative records and direct follow-up contact with families to ascertain outcomes. However, several limitations require acknowledgement. This study is retrospective and limited by a relatively small sample size, which reduces statistical power to detect modest differences between techniques. Follow-up was a minimum of six months, but longer surveillance is necessary to capture late recurrences or complications. Selection bias is possible, as surgeon preference and emergency presentation influenced technique choice in some cases.

In summary, the available evidence, including our present series, indicates that both LICS and LAPIRS are safe and effective for paediatric inguinal hernia repair, each with distinct advantages. LAPIRS offers technical simplicity, shorter learning curves, and potentially superior cosmesis, whereas LICS remains a robust intracorporeal solution when indicated. Future prospective multicentre randomised studies with standardised outcome measures and extended follow-up are needed to determine whether clinically meaningful differences in recurrence, complications, cosmesis, or cost exist between these approaches.

## Conclusions

Laparoscopic repair of paediatric inguinal hernia is a safe and effective alternative to open repair, with the added benefit of contralateral exploration and excellent cosmetic outcomes. In this retrospective cohort study, both LICS and LAPIRS demonstrated comparable perioperative and postoperative results. Operative times were similar between techniques, and neither was associated with intraoperative complications or recurrence during follow-up. Morbidity was minimal, limited to a single case of umbilical disfigurement after LAPIRS. While international literature suggests that LAPIRS may offer advantages in terms of ease of learning, reduced operative time, and superior cosmetic outcomes, our study supports the conclusion that both approaches are equally feasible and reliable in routine practice. The absence of significant differences in outcomes underscores that surgeon expertise and institutional preference may reasonably guide technique selection.

Given the limited sample size and retrospective design, further prospective multicentre studies with larger cohorts and longer follow-up are warranted. Such research will be critical in clarifying potential differences in recurrence, cost-effectiveness, and long-term cosmetic satisfaction, thereby informing best practices for paediatric inguinal hernia repair.
